# The antimicrobial molecule trappin-2/elafin has anti-parasitic properties and is protective *in vivo* in a murine model of cerebral malaria

**DOI:** 10.1038/srep42243

**Published:** 2017-02-09

**Authors:** Christian Roussilhon, Gilles Bang, Fabien Bastaert, Brigitte Solhonne, Ignacio Garcia-Verdugo, Roger Peronet, Pierre Druilhe, Anavaj Sakuntabhai, Salaheddine Mecheri, Jean-Michel Sallenave

**Affiliations:** 1 Unité de génétique fonctionnelle des maladies infectieuses and CNRS Unité de recherche associée 3012; Paris, 75015, France; 2 Unité de Défense Innée et Inflammation, Institut Pasteur, 25 rue du Dr Roux, Paris, 75015, France; 3 INSERM U874, Institut Pasteur; 4 INSERM U1152, Faculté de Médicine site Bichat, Université Paris Diderot, Université Sorbonne Paris-Cité, 16, rue Henri Huchard, Paris, 75018, France; 5 Institut Pasteur, Unité de Biologie des Interactions Hôte Parasites, CNRS ERL9195 and INSERM U1201, Paris F-75015, France; 6 CNRS ERL9195 and INSERM U1201, Paris F-75015, France; 7 INSERM U1201, Paris F-75015, France; 8 TheVac4all initiative, Institut Cochin, Paris, France

## Abstract

According to the WHO, and despite reduction in mortality rates, there were an estimated 438 000 malaria deaths in 2015. Therefore new antimalarials capable of limiting organ damage are still required. We show that systemic and lung adenovirus (Ad)-mediated over-expression of trappin-2 (T-2) an antibacterial molecule with anti-inflammatory activity, increased mice survival following infection with the cerebral malaria-inducing *Plasmodium berghei* ANKA (*Pb*ANKA) strain. Systemically, T-2 reduced *Pb*ANKA sequestration in spleen, lung, liver and brain, associated with a decrease in pro-inflammatory cytokines (eg TNF-α in spleen and lung) and an increase in IL-10 production in the lung. Similarly, local lung instillation of Ad-T-2 resulted in a reduced organ parasite sequestration and a shift towards an anti-inflammatory/repair response, potentially implicating monocytes in the protective phenotype. Relatedly, we demonstrated *in vitro* that human monocytes incubated with *Plasmodium falciparum-*infected red blood cells (*Pf*-iRBCs) and IgGs from hyper-immune African human sera produced T-2 and that the latter colocalized with merozoites and inhibited *Pf* multiplication. This array of data argues for the first time for the potential therapeutic usefulness of this host defense peptide in human malaria patients, with the aim to limit acute lung injury and respiratory distress syndrom often observed during malaria episodes.

Current estimates by the World Health Organization (WHO) indicate that every year global deaths due to malaria range between 1 and 2 million people with 80% of deaths occurring among children less than 5 years of age and living in Sub-Saharian Africa^1^,^2^,^3^. Compared to uncomplicated malaria, cerebral Malaria (CM) is a severe clinical manifestation associated with a mortality rate of up to 20–30%^4^. Although the direct link between *Plasmodium-*infected red blood cells (*Pf*-iRBCs), sequestration to the brain endothelium and CM is still a matter of debate^5^,^6^, there is little doubt that CM is the result of over-zealous inflammatory reactions not only in the brain but also in other key organs such as the spleen and the lung^7^,^8^,^9^,^10^,^11^, leading to major complications such as coma and seizures.

The lethal murine malaria model of *Plasmodium berghei* ANKA (*Pb*ANKA) has mainly been used to study Experimental Cerebral Malaria (ECM)^12^, and several studies have shown that in mice, ECM pathology also results from an exacerbated pro-inflammatory immune responses, including the sequestration of monocytes, CD8+ and CD4+ T cells in the organ capillaries^13^,^14^,^15^,^16^,^17^.

Pulmonary complications such as acute respiratory distress syndrom (ARDS) have been shown to occur during malaria, often in association with cerebral manifestations^7^,^11^,^18^,^19^,^20^,^21^, but the lung has attracted considerably less attention than other organs in the pathogenesis of this disease^12^,^21^,^22^,^23^,^24^,^25^,^26^. Mechanistically, the lung-associated pathology probably arises from CD36-dependent sequestration of iRBCs at the alveolar-capillary barrier, followed by pro-inflammatory cytokine production, neutrophil and monocyte activation, and sequestration of the cells at the alveolar-capillary endothelial capillary barrier, ultimately leading to barrier rupture and lung failure^7^.

We and others have shown that systemic and local mucosal anti-protease expression can be modulated in these conditions^27^,^28^. In particular, trappin-2 (abbreviated T-2 for the rest of the manuscript), is a molecule which has been shown to display pleiotropic antiprotease/anti-inflammatory and anti-microbial activities against a range of pathogens, including bacteria, viruses, and fungi^29^,^30^,^31^. Therefore, given the currently anti-microbial and anti-inflammatory described properties of the T-2 molecule, we hypothesized that over-expression of this mediator may be beneficial *in vivo* in a murine model of *Pb*ANKA infection. To that aim, since wild type (WT) mice are natural ‘knock-out’ for the T-2 molecule^32^, a system of T-2 over-expression by recombinant adenovirus vectors (Ad) expressing human T-2 was used in the present study. We showed here that T-2 is an efficient antimalarial both *in vitro* and *in vivo,* and can prevent maladaptive inflammation and promote tissue-repair, making this molecule a potentially important tool in severe and complicated malaria.

## Results

### T-2 confers protection against ECM in mice

**Figure 1:**
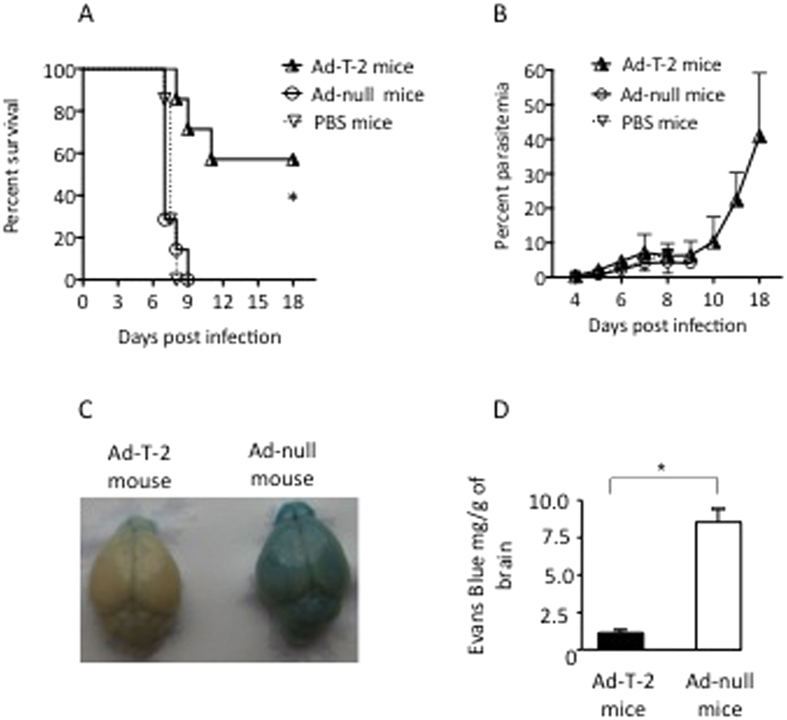
Survival and parasitemia in i.p Ad-T-2-treated mice following i.p *Pb*ANKA infection. (**A**) increased survival after Ad-T-2 treatment of PbANKA-infected mice. A representative result of 3 distinct experiments is shown. In each experiment, C57BL/6 WT mice were inoculated i.p either with PBS (n = 7), or with a dose of 10^8^ pfu of Ad-null (n = 7) or Ad-T-2 (n = 7). 48 hrs later, 10^5^ GFP-transgenic *Pb*ANKA (MR867) iRBCs were given i.p and mouse survival was followed daily for up to 18 days. Results were plotted using Kaplan-Meier plot and analyzed by log-rank test. *Indicated statistical significance (p < 0.05). **(B**) Blood parasitemia from the experiment shown in A) was determined by FACS analysis, by measuring GFP fluorescence of GFP-transgenic *Pb*ANKA, as indicated in Methods. (**C,D**) Protection against brain damage in Ad-T-2-treated mice. (**C**) Macroscopic observations of representative brains from Ad-T-2-treated (on the left) and Ad-null-treated mice (on the right) are shown. For this independent experiment, 5 mice per group were inoculated i.p with Ad-vectors, as in A) followed 2 days later with an i.p inoculation of 10^5^ iRBCs of the *Pb*ANKA strain. At day 5 post-infection, mice were injected retro-orbitally with 0.1 ml of 2% (wt/vol) Evans blue dye in PBS and 1 h later they were deeply anesthetized and perfused intracardially with 20 ml PBS. Brains were retrieved and photographed. (**D**) Evans blue dye extravasation measurements obtained by spectrophotometry. The absorbance of Evans blue that infiltrated brains was measured at 620 nm by comparison with a standard curve of Evans blue in formamide and results were expressed as mg of Evans blue per g of brain tissue. *Indicates a significant difference between groups (Student t-test, p = 0.018).

In mice inoculated with the lethal strain of Pb-ANKA, the percentage of surviving animals was markedly increased in Ad-T-2-pre-treated mice as illustrated in Fig. 1A (one of 3 representative experiments). Indeed, up to 60% of T-2 expressing mice survived while none survived in the control groups of WT PBS-treated mice or Ad-null treated mice (p value = 0.0104 by Log-rank tests). The course of *P. berghei* blood parasitemia was not significantly altered by T-2 (Fig. 1B) during most of the follow-up, when mice from the different groups were still alive, even if it was slightly and transiently contained by T-2 at early time points, before D4 (data not shown). Of note, there was no detectable brain damage of Ad-T-2 -protected mice compared to that of WT C57BL/6 mice in which leakage of Evans blue showed pathological alterations in the blood brain barrier permeability (Fig. 1C,D). Since there was no difference detectable between PBS and Ad-null treated mice (hence, no impact of Ad-null treatment per se), the following mechanistic experiments were carried out by comparing Ad-null and Ad-T-2 inoculated mice.

### *In vivo* assessment of T-2 expression in sera and mice tissues

**Figure 2:**
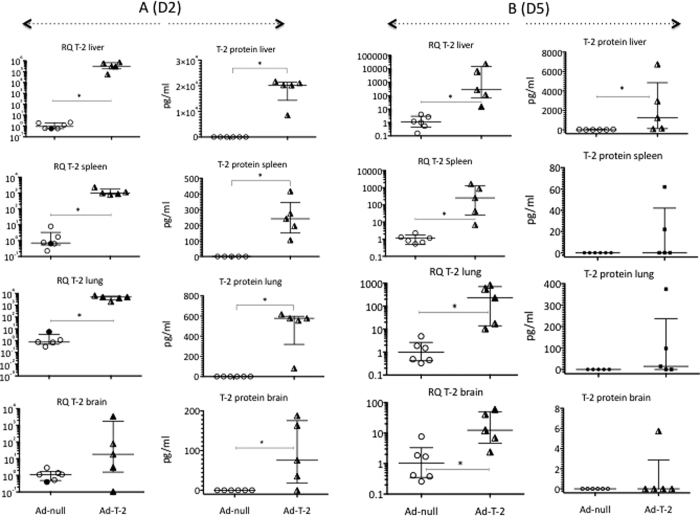
Quantification of tissue T-2 transcripts and T-2 protein levels at D2 (**A**) and D5 (**B**) post *Pb*ANKA infection. C57BL/6 mice were inoculated i.p at D0 with either 10^8^ pfu of Ad-null- or Ad-T-2, and they were treated two days later i.p with 10^5^ GFP-transgenic *Pb*ANKA. Two (D2, A) or five (D5, B) days later, T-2 mRNA levels (from left to right, columns 1 and 3) were measured by RT q-PCR in different organs: Rq ( = 2^−∆∆Ct^), using HPRT as the normalizer gene and ‘Ad-null + *Pb*ANKA’ as the reference treatment. In parallel, T-2 protein levels were measured by ELISA in tissue extracts of the same organs (columns 2 and 4). In each column, Ad-null and Ad-T-2 results were compared and *Indicates when median ( + /− inter-quartiles) values were found significantly different (Mann-Whitney test, p < 0.05).

To investigate the implication of T-2 in malaria pathogenesis, we determined its mRNA and protein levels following sequential i.p injections of Ad-T-2 and *Pb*ANKA. As expected, since WT C57BL/6 mice do not express the T-2 gene^32^, expression of T-2 mRNA or protein was absent in all the organs from Ad-null+ *Pb*ANKA–inoculated animals, either at Day2 (D2, Fig. 2A) or Day 5 (D5, Fig. 2B) post *Pb*ANKA infection (i.e 4 and 7 days post Ad- injection, respectively). By contrast, and in keeping with the abundant litterature reporting transgene expression following i.p. Ad-injection (eg ref 33), a robust and significant T-2 mRNA and protein expression was noted in the liver of mice D2 after *Pb*ANKA infection (Fig. 2A) and at a comparatively lower level at D5 (Fig. 2B). An intermediate T-2 expression level was measured in the lung and spleen, and weaker (and more variable) T-2 levels were present in the brain. From D2 (Fig. 2A) to D5 (Fig. 2B), T-2 expression was reduced about 1000 fold in all organs tested except in the brain where the amount of T-2 remained extremely low. Consistent with this expression pattern, high levels of serum T-2 were measured in the sera of Ad-T-2-treated animals at D2, and these levels tended to be markedly variable at D5 (Supplementary Fig. S1). As expected, no T-2 was detectable in the serum of Ad-null-treated mice at any time.

**Figure 3:**
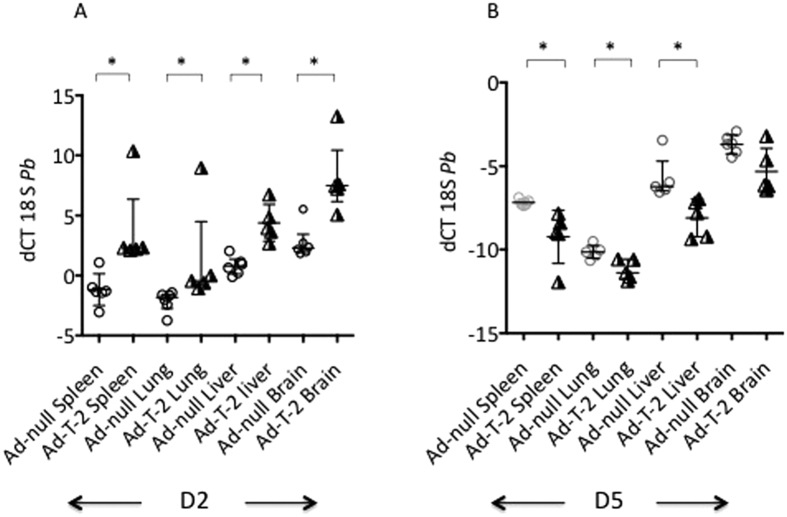
Quantification of tissue *Pb*ANKA 18 S mRNA levels at D2 (**A**) and D5 (**B**) post *Pb*ANKA infection. The same samples obtained for Fig. 2 analysis were assessed for *Pb*ANKA 18 S mRNA expression at D2 (A, left graph) and D5 (B, right graph) in the same organs. dCT = (CT 18 S minus CT HPRT) was used as a read-out for parasite load. Each individual symbol represents a different animal. Ad- T-2 + *P.b* and Ad-null + *P.b* were compared at each time point, and for each organ, and *Indicated when median ( + /− inter-quartiles) values were found significantly different (Mann-Whitney test, p < 0.05).

We then tested whether T-2 over-expression was associated with a modulation of the parasite burden in the same organs as above. For this purpose, the parasite load was assessed by q-PCR using the *Pb*ANKA 18 S as read-out. At D2 after *Pb*ANKA infection, regardless of treatment, higher levels of *Pb*ANKA 18 S were found in the lung and spleen, compared to the liver and brain (a low dCT value reflecting a high gene expression level as shown in Fig. 3A). Notably, at D2 (Fig. 3A) but not at D5 (Fig. 3B) Ad-T-2 -treated animals had a lower parasite burden (higher dCT) in all organs, compared to Ad-null-treated controls, correlating with higher T-2 expression levels. In contrast, at D5, at a time when T-2 expression was drastically decreased, the parasite load dramatically increased in all organs (negative values, Fig. 3B). These data suggest that the parasite burden is directly controled by the level of T-2 expression in the tissues and that the rebound of parasitic load at D5 is consistent with the transient expression of Ad-mediated T-2.

### Relationship between T-2 expression and cytokine responses *in vivo*

We then assessed by q-PCR whether the differential parasite burden measured in different organs was associated with particular patterns of inflammatory cytokine expression. When Ad-T-2- and Ad-null-treated mice followed by *Pb*ANKA infection were compared, T-2 treatment was associated with a marked anti-inflammatory effect at D2 (Table 1, Day 2), as indicated by a remarkable up-regulation of IL-10 and a simultaneous down-regulation of TNF-α, MIP-1α, KC in the lung, and of TNF-α, IL-1β, IL-6, IFN-γ, MCP-1 and KC in the spleen. In addition, in the lungs, there was a trend for decreased MIP-2 levels at D2 in Ad-T-2-treated mice. Down regulation of TNF-α (and a trend towards that of IFN-γ) were also observed in the brain of Ad-T-2-treated mice (Table 1, Day 2). Of note, in these animals, the expression of the anti-inflammatory cytokine IL-10 was sustained at D5 in the lungs. Irrespective of the experimental treatment, IL-10 expression was absent in the brain at D2, and only became measurable in this organ at D5 (Table 1, Day 5). At that same time point, significantly elevated levels of TNF-α and IL-1β (in the lung), IL-6, MCP-1, as well as to a lesser extent TNF-α and IFN-γ (in the liver), were detected in Ad-T-2-treated mice, when compared to Ad-null-treated mice (Table 1, Day 5). These data indicate that at early time points of the infection where Ad T-2 is highly expressed in the organs, the inflammatory response was kept globally under control and this was associated with protection against ECM.

### Intranasal instillation of T-2 has long-range and systemic effects on PbANKA infection

As shown above, at D2 post *Pb*ANKA inoculation, the lung and the spleen were the organs where parasites were massively sequestered (Fig. 3A) and where i.p-mediated Ad-T-2 delivery mostly promoted an anti-inflammatory phenotype (Table 1, Day 2). We therefore reasoned that expressing T-2 locally in the lung, an easily accessible organ, during malaria infection, could have a similar protective effect. Hence, Ad-vectors were given intra nasally (i.n), followed by i.p *Pb*ANKA inoculation two days later, and mouse survival and biological measurements were performed at D2 (i.e. 4 days after Ad inoculation).

**Figure 4:**
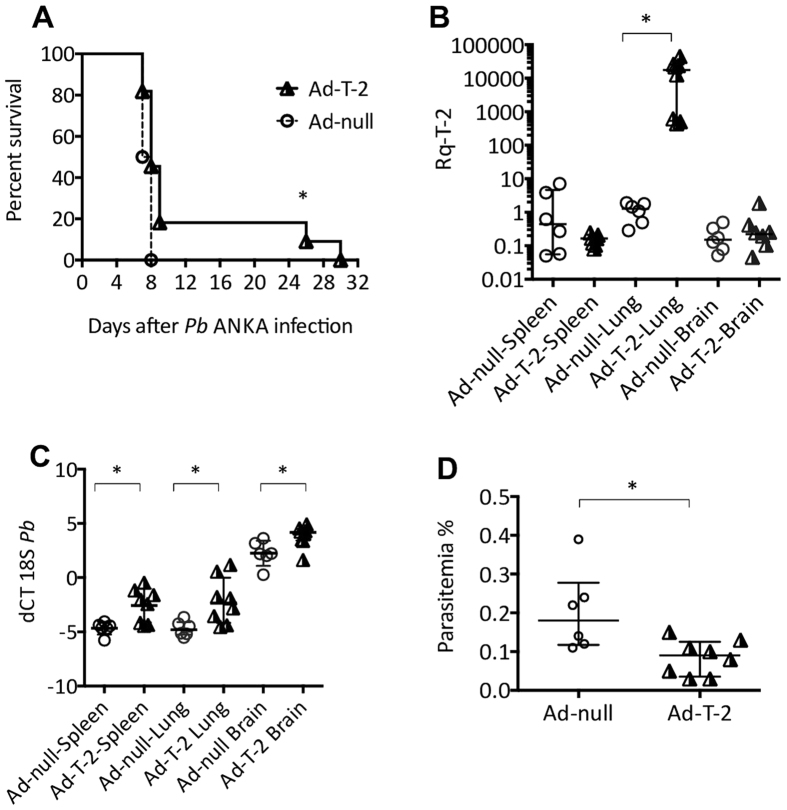
Sequential i.n Ad-null/Ad- T-2 and i.p *Pb*-ANKA administrations. (**A**) C57BL/6 mice treated i.n with 4.5 × 10^8^ pfu of Ad-null (n = 7) or Ad-T-2 (n = 5) were inoculated 48 hrs later i.p with 10^5^ GFP-transgenic *Pb*ANKA and differences in survival were determined between the two groups of mice. Results were plotted using Kaplan-Meier plot and analyzed by log-rank test and *denotes statistical significance (p = 0.039). (**B**–**D**) C57BL/6 mice were treated i.n with 4.5 10^8^ pfu of Ad-null (n = 6) or Ad-T-2 (n = 8). 48 hrs later, they were inoculated i.p with 10^5^ GFP-transgenic *Pb-*ANKA. 2 days (D2) post *Pb-*ANKA administration, organs (spleen, lung and brain) and sera were recovered for q-PCR measurements of T-2 mRNA (B: Rq was measured as explained in Table 1 legend), *Pb-*ANKA 18 S mRNA (C: dCT measured as in Fig. 3) and for blood parasitemia count (D: measured as explained in Methods). *Indicates when median (+/− inter-quartiles) values were found significantly different (Mann-Whitney test, p < 0.05).

With regard to mouse viability (Fig. 4A), there was increased survival in the i.n. Ad-T-2-treated group, although less impressive when compared to the i.p protocol of Ad-delivery (Fig. 1). Nevertheless, all Ad-null-treated animals died within 8 days, whereas 20% of Ad-T-2-instilled mice survived the critically acute episode of ECM (*p* = 0.039 by Log rank test) after which they died of anemia.

In Ad-T-2 treated animals, at D2 post *Pb*ANKA inoculation, T-2 mRNA expression was found to be maximal in the lung, but barely detectable in other organs, demonstrating the lung compartimentalization of Ad-treatment (Fig. 4B). T-2 protein was detected in serum samples at significant levels (median with 25% and 75% percentiles: 156 [138–171] pg/ml), albeit at a concentration 24.5 fold lower than when Ad-T-2 was given i.p. (Supplementary Fig. S1). We then tested whether following i.n instillation, T-2 over-expression was, as with the i.p treatment, associated with a differential parasite burden locally in the various organs. The highest parasite load was found in the spleen and lung as shown in Fig. 4C (a low dCT value reflecting a high gene expression level) and that load was comparatively limited in mouse brains. Critically, i.n Ad-T2 treated animals showed reduced parasite levels in each of the organs considered, even distantly from the lung such as the spleen and the brain (Fig. 4C). When parasitemia was measured, and in line with the trend observed in the previous experiments where Ad-T-2 was instilled i.p, a 2.4 fold decrease in the number of circulating parasites was observed at D2 in i.n Ad-T-2 treated animals, compared to the Ad-null control group (Fig. 4D).

**Figure 5:**
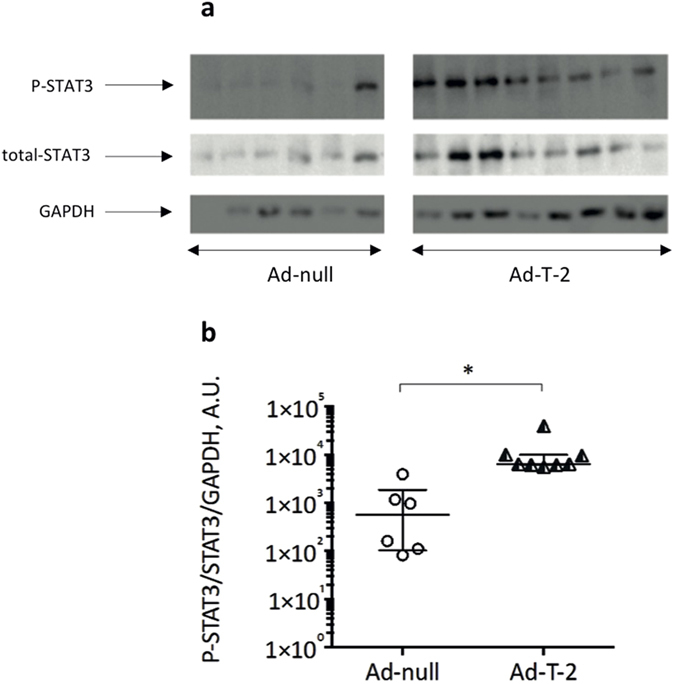
Determination of lung STAT3 expression and activation following sequential i.n Ad-null/Ad- T-2 and i.p *Pb*-ANKA administrations. The lung samples (each one representing a different animal) obtained for Fig. 4 analysis were also assessed for expression of total STAT3, phosphorylated (P)-STAT3 and GAPDH. Lung extracts were analysed by SDS-PAGE, followed by Western Blot analysis. The PVDF membrane was incubated with rabbit anti-STAT-3 or anti-P-STAT3 antibodies (diluted 1:2,000) whereas anti-GAPDH antibody (to check for equal loading) was diluted 1:10,000. After incubation with secondary antibodies, the membrane was developed as described in Methods. The gel presented is a cropped version of the original gels presented in Fig. 3 Supplementary. (**B**) The ratios of P-STAT3/STAT3/GAPDH were determined (A.U = arbitrary units) and used as an indication of STAT3 activation. *Indicates a significant difference between groups (Student t test, p = 0.0007).

When cytokine levels were measured at D2, MIP-1α, MCP-1, KC, IFN-γ, TNF-α, IL-1β and IL-6 levels were significantly reduced in the spleen of Ad-T-2 -treated mice compared to Ad-null-treated mice (Table 2). In the lung, by contrast, both IL-10 and MCP-1 levels were strikingly increased whereas KC and MIP-2 expression was significantly down-regulated upon Ad-T-2 instillation (Table 2). Because IL-10 and MCP-1 have been both shown to activate STAT-3^34^,^35^,^36^,^37^, expression of this transcription factor was assessed in total lung extracts, and T-2 expression was found to be associated with a strong STAT-3 up-regulation in the lung (Fig. 5A and B).

Importantly, IL-10, MCP-1 and STAT-3 are part of a network promoting lung repair^34^,^35^,^36^,^37^ and cellular homeostasis^38^. In the present rodent model of lethal malaria infection, the activation of this network was associated with a significant reduction in circulating creatine kinase (CK) and alanine aminotransferase (ALT) levels, 2 systemic markers of cellular disturbance and liver damage occuring frequently during malaria^39^,^40^. Specifically, the levels of CK and ALT were significantly reduced 1.7 and 1.6 fold respectively in Ad-T-2 i.n. compared to Ad-null i.n. –treated mice (Supplementary Fig. S2).

### T-2 secreting human monocytes inhibit *P. falciparum* malaria growth *in vitro*

In mice, following *Plasmodium* infection, monocytes have been shown to be sequestered in the capillary beds of the lungs, brain, and spleen^10^. Because T-2 expression was shown *in vivo* to modulate the parasite load (Figs 3A and 4B) and to simultaneously promote a strong expression of the monocytic chemotactic factor MCP-1 following instillation in the lung (Table 2), we wondered whether monocytes could mediate some of the protective responses associated with T-2 expression.

**Figure 6:**
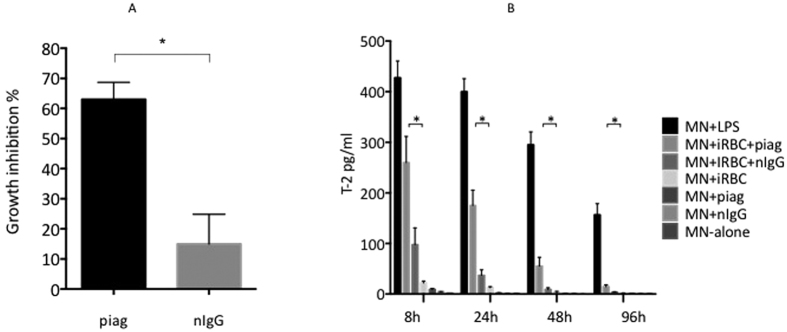
ADCI assays and secretion of T-2 by human monocytes. (**A**) ADCI assays were carried out in 48 well culture plates by co-incubating 2 × 10^5^ monocytes (MNs) with 2 mg/ml of IgGs from either malaria immune (PIAG) or malaria naïve (nIgG) donors and *Pf*-iRBCs were added at 0.5% starting parasitemia (and 2.5% hematocrit). After 96 hrs of co-culture, parasitemia was determined by FACS analysis. Assays were performed in triplicates and *Indicate statistical significance when comparing the effect of PIAG to that of nIgG.(**B**) MNs were left untreated or incubated with either LPS (positive controls), human IgG alone (2 mg/ml of either nIgG or PIAG), *Pf*-iRBCs alone or *Pf*-iRBCs + IgGs (nIgGs or PIAG) in 48 well culture plates over a 96 hr time course period. Concentrations of T-2 in co-culture supernatants were determined by ELISA at 8 h, 24 h, 48 h and 96 h. Assays were performed in triplicates and *Indicate statistical significance (Student-t test, when comparing MNs + *Pf*-iRBCs + PIAG with MNs + *Pf*-iRBCs + nIgGs.

Given that WT mouse monocytes do not express T-2, we used the antibody-dependent cell inhibition assay (ADCI) to determine whether human monocytes (MNs) produced T-2 when these cells were stimulated following incubation in the presence of *Pf*-iRBCs and IgGs purified from a Pool of Immune African Globulins (PIAG). In accordance with previous work^41^, Fig. 6A shows that MNs co-incubated with PIAG (but not with non immune IgGs, nIgGs) displayed high ADCI activity by efficiently restricting parasite multiplication *in vitro*. This effect was correlated with the secretion of T-2 by purified MNs cocultured with iRBCs and PIAG (Fig. 6B). This secretion was drastically reduced when PIAG was replaced by nIgGs demonstrating that the presence of malaria hyper-immune IgG was necessary for up-regulating the production of parasite inhibitory factors, notably T-2. By comparison, and although significant compared to MNs alone, MNs incubation with *Pf*-iRBCs (but without immune antibodies) induced a very small amount (but still measurable) of T-2 protein, indicating nonetheless that infected iRBCs by themselves can constitute a ‘danger signal’ able to induce T-2 secretion, even in the absence of parasite-specific antibodies.

### Immunolocalization of T-2 in iRBCs

**Figure 7:**
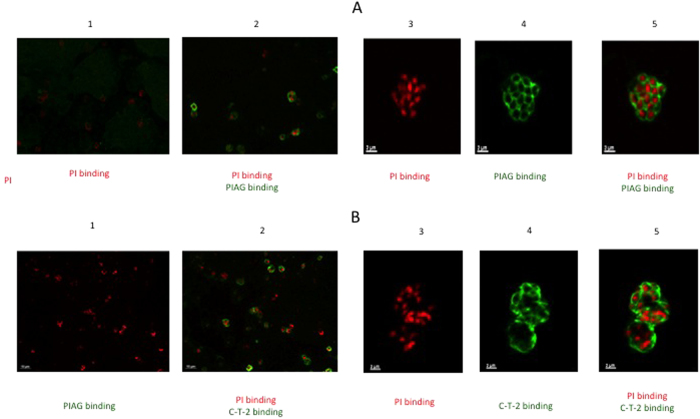
Illustration of C-T-2 and PIAG colocalization with merozoites. (**A**) Co-localization of parasite immune IgGs (PIAG) with merozoites. Merozoite bags were prepared as indicated in Methods and incubated or not with PIAG. Nuclei were identified with PI binding (red fluorescence, low and high magnification, panels 1–2 and 3–5, respectively). Binding of PIAG with merozoites (green fluorescence) is shown both at low (panel 2) and high (panels 4–5) magnification. (**B**) localization of C-T-2 within merozoites. Merozoite bags were incubated with 10 μM of recombinant human C-T-2 (aa 38–95 from the full length T-2 molecule), as indicated in Materials and Methods. After permeabilization with acetone/methanol (50:50 dilution), rabbit anti-T-2 IgG (1:50 diution) and goat anti-rabbit IgG (1:500 dilution) coupled with Alexa 488 (green fluorescence) were added sequentially and incubated for 1 hr. As above, nuclei were identified with PI binding (red fluorescence, low and high magnification, panels 1–2 and 3–5, respectively). Binding of C-T-2 on merozoites (green fluorescence) is shown both at low (panel 2) and high (panels 4–5) magnification.

Because coculture of MNs with PIAG in the presence of iRBCs induced the secretion of T-2 (Fig. 6B), and because these parasite-specific antibodies have been previously shown to recognize merozoite surface antigens (among others, MSP-3, GLURP, SERA/SERP and MSP-1 block 2 antigens^42^), we hypothesized that T-2 may have an effect on parasite multiplication through interaction with merozoites. Indeed, we confirmed by immunofluorescence that the PIAG used in our experiment colocalize with merozoites (Fig. 7A) and that recombinant C-T-2 (see below) also immuno-localized with the parasitophorous vacuole membrane of *Pf.* schizonts (as shown when using merozoite “bags”, Fig. 7B).

### Direct inhibition of *in vitro* parasite multiplication by T-2, C-T2 (elafin) and N-T2

**Figure 8:**
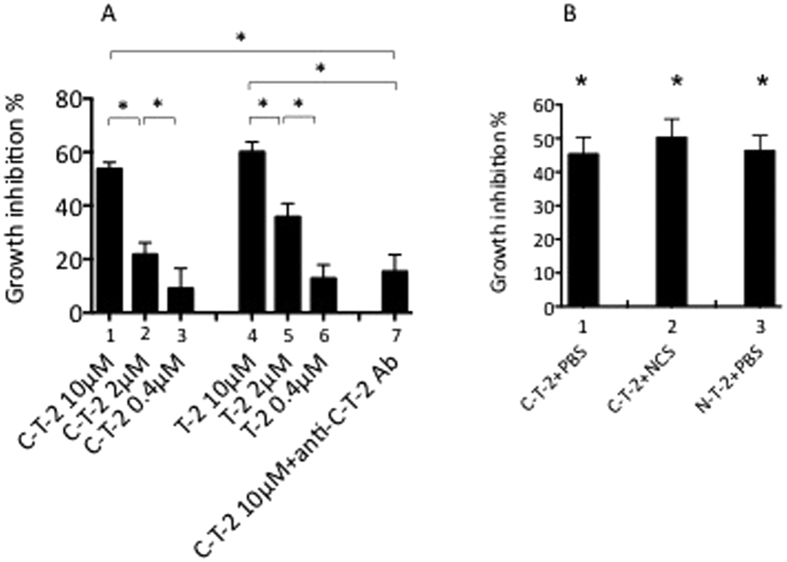
Antiparasitic activity of T-2, C-T-2, and N-T-2 molecules. (**A**) Both full-length T-2 and C-terminal T-2 dose-dependently inhibit *P. falciparum* growth. Asexual *Pf*-iRBCs blood stages were incubated for 96 hrs (at 37 ^o^C and in a 5% CO_2_ athmosphere) with either human T-2 (the 95 aa full length T-2 molecule) or C-T-2 (a.a 38–95 of T-2) at a final concentration of 0.4 to 10 μM. Specificity of C-T-2 activity was confirmed by incubating C-T-2 (10 μM) with a blocking antibody (Ab) against C-T-2 (1:50 dilution) prior to exposure to *Pf*-iRBCs (histogram 7). Assays were done in triplicates and parasitemia was measured as explained in Methods. All treatments showed a statistically significant difference (not shown) compared to untreated control *Pf-*IRBCs (100% parasite growth). Statistical significances between each dose of the two dose-responses and between histograms 1 and 7 are indicated (Student t- test, *p < 0.05). (**B**) The anti-protease activity of C-T-2 is not necessary for its anti-Plasmodium function. The same assay as in Fig. 8A was performed with N-T-2 (aa 1–50 from the T-2 molecule at 6 μM on, histogram 3). Alternatively, C-T-2 (6 μM final concentration) pre-treated with either PBS (histogram 1) or with the oxidant molecule NCS (to inactivate chemically its antiprotease activity, histogram 2) was used in the assay. *Indicating statistical differences (Student t-test, p < 0.05), compared to the control *Pf*-iRBCs alone (100% parasite growth).

As a result of this immuno-localization, and given that T-2 treatment decreased the initial Pb-ANKA parasite levels *in vivo,* we investigated whether T-2 *per se* could inhibit *in vitro* the multiplication of iRBCs. Compared to untreated *Pf-*iRBCs, we found that the 9.9 kDa T-2 full length molecule (aa 1–95, net cationic charge of +7, Fig. 8A, histograms 4–6), as well as the 6.0 kDa C-terminal T-2 (also known as elafin and composed of T-2 a.a 38–95, net cationic charge of +3, Fig. 8A, histograms 1–3) were able, in a dose-dependent fashion, to significantly inhibit *Pf* multiplication *in vitro*. The specificity of this effect was demonstrated by using a polyclonal rabbit blocking antibody raised against T-2, which significantly prevented the parasite growth inhibition effect by 70.6% (Fig. 8A, compare histograms 1 and 7.

In addition, because both full length T-2 and C-T-2 possess the antiprotease site of the T2 molecule (Ala62- Met63 representing the scissile bond P’1-P1), we set up to determine whether antiprotease activity, cationic charges (or both), were responsible for the antiparasitic activity. For that purpose, the antiprotease site of C-T-2 was inactivated chemically (through oxidation by N-chlorosuccinimide/NCS). Similarly, N-T-2, a chemically-synthetised T2 moiety (aa 1–50, inactive as an antiprotease^43^, with a net cationic charge of +5) was also used. Remarkably, all T-2 moieties had significant, and similar antiparasite activities, when compared to untreated controls (Fig. 8B), suggesting that cationic charges, and not the antiprotease activity, contribute to the T-2/C-T-2/N-T-2 antiparasitic functions.

## Discussion

We demonstrate here for the first time that trappin-2 (T-2), a cationic antimicrobial/anti-inflammatory agent^29^,^30^,^31^,^32^,^43^ is an efficient inhibitor of malaria parasite growth and a critical regulator of the pathophysiological consequences of asexual blood stage parasite infection in a rodent model of ECM. This was demonstrated *in vitro* with the observation that direct interaction between various T-2 moieties (T-2, N-T-2, and C-T-2/elafin) significantly hampered both *P. falciparum* and *P. berghei* parasite multiplication in culture conditions. Importantly, although both T-2 and C-T-2 have antiprotease activities^27^,^29^,^30^,^43^, we showed that the latter was not the mechanism involved here, since C-T-2 whose antiprotease activity had been chemically inactivated, was still able to efficiently inhibit *Plasmodium* multiplication (Fig. 8A). Similarly, N-T-2, which does not bear the antiprotease site, was as inhibitory as the other molecular entities (Fig. 8B). The fact that the antiprotease activity was not involved in the antimalarial activity described here is reminiscent of our previous findings and those of others demonstrating firstly that N-T-2 can kill *P. aeruginosa* and *S. aureus*^43^, and secondly that a genetically-engineered T-2 mutated for its antiprotease activity was as efficient as the parent molecule in killing *P.aeruginosa, S.aureus, E.Coli* bacteria, as well as in killing *A.fumigatus* and *C.albicans* fungi^44^.

Importantly, all 4 molecules T-2, N-T-2, C-T2, inactive C-T2 are cationic (with net positive charges of +7, +5, + 3, +3, respectively), suggesting that as for other AMMs, this property is of paramount importance for the anti-*Plasmodium* activity described here.

In line with our previous observations, there was no direct relationship between blood parasitemia level and disease severity in this mouse model of ECM^45^, but we show in the present study that following *Pb*ANKA infection, iRBC sequestration occurred in all studied tissues, with a decreasing gradient from the spleen and the lung, to the liver and the brain. In addition, a direct link between the parasite loads detected in these various organs and the production of several pro-inflammatory cytokines (eg, TNF-α and IFN-γ among others) was observed (Table 1). Mechanistically, i.p-instilled Ad-T-2 was found associated with immunodulatory effects in the spleen and the lung D2 after *Pb*ANKA injection. Remarkably, at that time point, there was an increase in lung IL-10 mRNA with that treatment which tended to persist up to D5. In contrast, proinflammatory cytokines mRNA levels (TNF-α, IL-1β, Il-6 and IFN-γ) were increased at D5 in i.p-instilled Ad-T-2 mice illustrating a balance in the timing of anti- and pro-inflammatory cytokine induction. Of note, the increase in proinflammatory cytokines observed at D5 occurred at a time when the level of mRNA coding for elafin was drastically decreased as a likely consequence of the transient expression of the transgene associated with the elimination of transduced cells^46^. Notwithstanding this, the early timing and intensity of T-2 expression was obviously adequate in providing protection from the acute episode of ECM.

When Ad-T-2 was given i.n. to C57Bl/6 WT mice, there was increased survival after *PbANKA* infection and simultaneously, reduced lung organ injury (also observed by histological methods, not shown), as assessed by the amount of circulating CK and ALT (Fig. S2). The fact that i.n Ad-T-2 administration was relatively less efficient than i.p instillation at protecting against death could potentially be explained by the lower level of systemic T-2 protein release that resulted from the i.n mode of administration. This is in keeping with the accepted notion of lung compartmentalization of i.n. Ad-mediated gene transfer, which requires protein spillover from the lung into the circulation, in order to achieve systemic levels. Because, as demonstrated by Lovegrove *et al*.^7^. lung injury occurs relatively late in *Pb*ANKA-infected C57BL/6 mice (i.e. 6–7 days after malaria infection), achieving a better protection may have required to induce persistent T-2 lung expression throughout the duration of the experiment, which was not possible with this i.n protocol. Still, using this methodological approach, the parasitic load was reduced in the lung (Fig. 4) and both IL-10 and MCP-1 levels were significantly upregulated locally in this organ (Table 2). Simultaneously, pro-inflammatory cytokines (MIP-1α, IFN-γ and IL-1β) were remarkably down regulated distantly in the spleen, showing encouragingly, and for the first time to our knowledge, that protecting the lungs (albeit probably with improved protocols) could have a positive clinical outcome in ECM studies.

Also, IL-10 is well known for its anti-inflammatory effect and has been shown to be critical in modulating inflammation in severe malaria^47^,^48^. Interestingly, using the *P. berghei* NK65 strain which induces acute respiratory syndrome without cerebral complications and which allows for a wider time window to study pathological consequences, van den Steen *et al*.^8^. showed that i.p dexamethasone treatment had some modulatory effects, inducing notably a decrease in the number of infiltrating CD8 T cells in the lungs, whereas similarly to our data, the lung IL-10 content was markedly increased.

In addition to IL-10, and as mentioned above, MCP-1 was also significantly upregulated in the lungs of i.n. Ad-T-2 treated mice (Table 2). Both IL-10 and MCP-1 have been shown to activate STAT-3, which is considered as a critical transcription factor involved in lung repair^34^,^36^. We show here for the first time that Ad-T-2-mediated over-expression is associated with STAT-3 up-regulation in the lung and may mediate some of the protective effects of IL-10 and MCP-1 in that organ. The latter has been extensively studied in lung infectious/inflammatory models and shown to be protective and to have immuno-regulatory properties^34^,^35^,^37^. In this context, migrating monocytes recruited to the spleen were found to be involved in the control of blood stage malaria, in CCR2-dependent fashion^49^. Whether MCP-1 has a direct protective effect on the lung in our model (through the promotion of tissue repair), or an indirect one, through monocyte chemotaxis, is difficult to assess, but it is tempting to speculate that the latter function may be critical through an ADCI mechanism. Indeed, several epidemiological studies have demonstrated that cytophilic immunoglobulin subclasses (IgG1 and IgG3), which are key effectors of ADCI, are intimately associated with the control of *Pf*-iRBCs multiplication *in vitro*, and with long-lasting protection against malaria attack *in vivo*^41^,^50^. Adoptive transfer of parasite-specific antibodies was shown to be protective in humans and although the specificity of some of these antibodies has been determined (such as those against MSP-3, GLURP, SERP, MSP-1 block2 for example), the precise nature of the monocyte-derived factors able to exert a parasiticidal effect have not been characterized so far. We suggest here, for the first time, that T-2 may be one of these critical monocyte-derived mediators involved in the control of parasite growth (Figs 6–8).

In conclusion, we showed that T-2 which is recognized in humans as an antimicrobial molecule and was previously shown to be efficiently active against bacteria and fungi^29^,^43^,^44^ is also capable, both *in vitro* and *in vivo*, to limit *Plasmodium* multiplication and is highly effective against the deleterious pathological consequences of *Pb*ANKA infection in ECM susceptible mice. Notably, our prophylactic model, where T-2 was given prior to *Plasmodium* infection, will have to be complemented with a therapeutic approach, where the order of the administrations is reversed. Notwithstanding the above, we believe that the pleiotropic nature of the human T-2 activity, such as direct antimicrobial as well as anti-inflammatory and tissue-repair promoting, makes it a potentially valuable candidate in the therapeutic armamentarium^51^,^52^,^53^, especially in severe and complicated malaria, where the effect of antimalarial drugs could be complemented by this natural compound.

## Methods

### Ethic statement

Procedures and experiments involving mice were approved by Institut Pasteur Safety Committee and performed in accordance with French legislation in general and in particular with Institut Pasteur Ethical Committee guidelines for animal handling (Approval Number A7515-27). Human blood samples from healthy malaria volunteers were sampled at the Etablissement Français du Sang (EFS, Paris) and used in accordance with French legislation in general and in particular with a convention between Institut Pasteur and EFS as licensed by Approval ID HS2003-3251.

### Adenovirus constructs

For *in vivo* Adenovirus (Ad)-mediated expression of human trappin-2 (h-T-2), the endogeneous leader and polyA sequences of the T-2 gene were inserted into the EI deleted region (replication-deficient) of Ad5 under the control of the mouse cytomegalovirus (MCMV), a potent promoter in rodents. Ad-MCMV-h-T-2 and Ad-dl70-3 constructs were amplified using established protocols^54^. Ad-MCMV-h- T-2 mice are called Ad-T-2 mice in the present manuscript and the Ad-dl70-3 control construct is referred as ‘Ad-null’, as it has no promoter, nor transgene.

### Animals

6–9 weeks old C57BL/6 mice were purchased from Janvier (Le Genest Saint Isle, France). All animals were maintained under 12 h-light/dark cycles and had free access to food and water and were maintained in the facilities of the Institut Pasteur for at least one week before the start of the experiments. All procedures were approved by institutional animal care committees and veterinary services (CETEA-2014–0009).

### Mice experiments

Six to nine-week-old “wild type” (WT) C57Bl/6 female mice (either untreated or receiving previously Ad vectors through the intra-peritoneal (i.p.) or intra-nasal (i.n.) routes) were inoculated i.p. with 10^5^ GFP-transgenic *Pb*ANKA iRBCs. Blood parasitemia was determined from the tip of the tail of each mouse, and blood samples were rapidly analyzed by FACS to determine individual parasitemia levels. Typically, in susceptible WT C57BL/6 mice, CM induction is characterized in our experiments by a gradual loss of activity, paralysis, ataxia, convulsions and sudden deep coma occurring 7 to 8 days after *Pb*ANKA inoculation. Individual mouse survival was checked on a daily basis, once a day during the first 5 days following parasite inoculation and at least twice a day thereafter.

PBS or Ad-vectors, at a dose of 10^8^ or 4.5. 10^8^ plaque-forming units (pfu), were administered intra-peritoneally (i.p.) or intra-nasally (i.n.) to WT C57Bl/6 female mice. Two days later, 10^5^ GFP-transgenic *Pb*ANKA iRBCs were inoculated i.p to each mouse. After a further 2 or 5 days period (referred to as D2 and D5 respectively), serum, liver, brain, spleen, and lungs were harvested and analysed for RNA and protein/cytokine expression.

### Measurement of blood brain permeability

C57BL/6 Ad-null and Ad-T-2 mice infected with *P. berghei* ANKA were injected retro-orbitally with 0.1 ml of 2% (wt/vol) Evans blue solution in PBS 5 days later. One hour after the PBS treatment, mice were perfused intracardially with 20 ml cold PBS under deep anesthesia (0.5 ml xylazine [Rompun], 1 ml ketamine [Imalgène 1000]), and brains were harvested and photographed. The quantification of Evans blue was determined in brains harvested and fixed in 2 mls 100% formamide (Merck) for 48 hrs at 37 °C. Absorbance was measured at 620 nm and compared with a standard curve of Evans blue in formamide. Results are expressed as microgram of Evans blue per gram of brain tissue.

### Cytokine and chemokine quantification by Real time quantitative PCR (RT-qPCR)

RNA samples were extracted from mice tissues (immediately frozen after organ isolation) with a Rneasy mini kit (Qiagen) as decribed by the manufacturer. RNA concentration was quantified with Nanodrop. Reverse transcription was performed on each RNA sample (1 μg) using the cDNA High-Capacity Archive kit from Applied Biosystems (Courtaboeuf, France) in a final reaction volume of 40 μl. Specific oligonucleotides (see below) were designed using the OLIGO Explorer software (Molecular Biology Insights, Inc., Cascade, CO). qPCR was performed with 40 ng cDNA, 300 nM of each primer and SYBR-Green PCR Master Mix (AbGene) to a final volume of 10 μl. Quantitative RT-qPCR measurements were performed on an ABI Prism 7900 Sequence Detector system (Applied Biosystems). After Taq activation (15 min), PCR cycles proceeded as follows: denaturation (15 s, 95 °C), annealing (30 s, 60 °C) and extension (30 s, 72 °C). When appropriate, the relative quantification (RQ) of mRNA levels was estimated by the ΔΔCt method using *HPRT* as normalizer gene, using the formula: RQ = 2^-ddCT^ with ddCT = dCT ‘test experiment’-dCT ‘control experiment’.

### Primer sequences selected for this work were as follows

HPRT-F: 5′-CAGGCCAGACTTTGTTGGAT-3′; HPRT-R: 5′-TTGCGCTCATCTTAGGCTTT-3′; T-2 –F: 5′-AAAGGTCCAGTCTCCACTAAGC-3′; T-2-R: 5′-CCTGGGCAGTCAGTATCTTTC-3′; KC-F: 5′-TCGTCTTTCATATTGTATGGTCA-3′; KC-R: 5′-CGAGACGAGACCAGGAGAAAC-3′; MIP1a-F: 5′-CTCCCTCCCAGTTGGTCAC-3′; MIP1a-R: 5′-AAAGGGCATATTTATTACTTCTCTG-3′; MIP2-F: 5′ TGGGTGGGATGTAGCTAGTTCC-3′; MIP2-R: 5′-AGTTTGCCTTGACCCTGAAGCC-3′; IFN-G-F: 5: GCG TCA TTG AAT CAC ACC TG-3′; IFN-g-R: 5 T CAC ACC TG-3GTTCC-3′; MIP2-R: 5′-AGTTTGC5′-AGGCGCTGTCATCGATTTCTC-3′; IL-10R: 5′-TGGCCTTGTAGACACCTTGGTC-3′; TNF-α-F: 5′-CTGTAGCCCAC-GTCGTAGC-3′; TNF-α-R: 5′-TTGAGATCCATGCCGTTG-3′; IL1-β-F: 5′-TTGACGGACCCCAAAAGAT-3′; IL1-β-R: 5′-GAAGCTGGATGCTCTCATCTG-3′; MCP-1-F: 5′-GGAAAAATGGATCCACACCTTG-3′; MCP-1-R: 5′-TCTCTTCCTCCACCACCATGCA-3′;

### Quantification of parasite loads in deep organs by real time quantitative PCR (RT-PCR)

Parasite loads were determined separately in each organ either at D2 or at D5 by using primers specific for the *Pb*ANKA 18S rRNA (F: 5′-AAGCATTAAATAAAGCGAATACATCCTTAC-3′ and R: 5′-GGAGATTGGTTTTGACGTTTATGTG-3′). Gene mRNA expression measured by RT-PCR was normalized to the endogenous control gene HPRT as explained above.

### Preparation of tissue extracts, protein measurement, Western Blot analysis

Organ tissues were rapidly isolated and homogenized with an Ultra Turrax device in RIPA buffer containing protease and phosphatase inhibitors (Roche). After further centrifugation to eliminate cell debris, supernatants were collected and protein content was dosed (Quick Start Bradford, Biorad), and assayed for T-2 concentration by ELISA. In parallel, SDS-PAGE (10% acrylamide) and Western Blot analysis for STAT-3 expression and activation was performed, using rabbit anti-STAT-3 and anti-P-STAT-3 antibodies (1:2,000 dilution in TBS-BSA) respectively (Cell Signalling). Antibody against GAPDH (Covalab) was used (1:10,000 dilution in TBS-BSA) to standardize protein loading. Secondary antibodies were anti-rabbit and anti-mouse IgGs (Jackson ImmunoResearch Laboratories, 1:20,000 dilution in TBS-BSA) respectively, both conjugated to horse raddish peroxidase. PVDF membranes were developed with the ECL2 Western Blotting substrate (Thermoscientific) in a PXI4 imager (Syngene).

### Purified proteins used for *in vitro* studies

Trappin-2 (T-2, aa 1–95) was obtained from Sigma. N-T-2 (aa 1–50 of the T-2 molecule) and C-T-2 (aa 38–95 of the T-2 molecule) were synthesized chemically, as explained in^43^. Inactive CT2 (devoided of anti-protease activity) was prepared by incubating C-T2 with the oxidant N-chlorosuccinimide (NCS) at a NCS/C-T2 molar ratio of 25 in PBS, as explained in^55^. After incubation for 30 min at room temperature, excess NCS was removed through 5 serial centrifugations in PBS buffer using Amicon filters (Amicon Ultracell 3k centrifugal filters). The final concentrated material (100 μl) was harvested and used for measurements in antiparasite growth assays (as explained below). Control C-T-2 and N-T-2 was also submitted to the same treatment, but NCS was replaced by PBS.

### Cytokine, chemokine and T-2 quantification by ELISA

Cytokine, chemokine and T-2 levels were tested in mice sera using R&D ELISA kits (for cytokines and chemokines) and an ‘in house’ ELISA, with an antibody raised against T-2, described in ref. 54.

### Parasite cultures

Asexual blood stage parasites of *Plasmodium falciparum* (3D7 clone) cultures were started with cryproserved and mycoplasma-free aliquots and using complete medium (CM) composed of RPMI 1640 with L-Glutamine (Invitrogen) supplemented with 0.36 mM Hypoxanthine, 35 mM HEPES, 23 mM NaHCO_3_ and 0.5% Albumax I (Invitrogen) in the presence of AB^+^ erythrocytes. Cultures were maintained at 37 °C in a humidified 5% CO_2_ incubator. Asexual blood stage parasites of *Pb*ANKA were cultivated for 24 hours with a rougly similar culture medium in which Albumax was replaced by FCS, 0.1% gelatin was added and the culture plates were agitated.

When particular asexual blood stage parasite morphotypes such as ring stages or schizont stages were required, cultures were treated either by 5% sorbitol (Acros Organics) for synchronisation at the ring stage or enriched at mature stages of schizogony by flotation on 1% porcine skin gelatin type A (Sigma-Aldrich).

Preparation of merozoite “bags” was performed after flotation of *Pf*-iRBC on 1% porcine skin for enrichment of mature forms. The enriched culture with mature schizonts was then incubated for 10 h (5% CO_2_, 37 °C) with 10 μM of the cysteine protease inhibitor l-transepoxy-succinyl-leucylamido-(4-guanidino) butane (E64)) in order to retain merozoites enclosed in the parasitophorous membrane (PVM) as PVM-enclosed merozoite structures. The preparation of merozoite bags was then delicately washed twice in CM and kept in culture at 37 °C until further use.

### Human blood monocyte isolation and preparation for functional assays

Peripheral blood mononuclear cells (PBMC) from healthy blood donors were separated by Ficoll-Hypaque (P.A.A. GmBH, Germany) density gradient centrifugation, washed in Ca^2+^ and Mg^2+^ free HBSS buffered with 10 mM HEPES (Invitrogen). Cells were frozen in heat-inactivated AB^+^ human serum with 10% DMSO (Sigma-Aldrich) at a final concentration of 15 × 10^6^ cells/ml and stored in liquid nitrogen until use. The phenotype of the fresh PBMCs and monocyte (MN) sub-populations were monitored by flow cytometry. MNs were isolated from PBMC with a human monocyte enrichment kit, without depletion of CD16 cells, according to manufacturer’s instructions (EasySep^®^, StemCell Technologies). The phenotypic analysis was performed with 10^6^ cells incubated in 100 μl of PBS-5% FCS (FACS buffer) in the presence of 0.1 mg/ml of anti-CD14-PE and anti-CD16-PC5 (Beckman Coulter) for 30 min at 4 °C in the dark. At least 5.10^4^ cells were acquired and then analysed by flow cytometry (FACSCalibur, BD Biosciences).

### Human Sera and IgG preparation

A pool of sera from 180 hyperimmune African adults gamma globulins (PIAG) previously found to confer passive protection when transferred to non-immune patients was used to purify the IgG preparation used herein. In addition, negative controls consisted of IgG prepared from a pool of plasma from French donors with no history of malaria (nIgG).

### Antiparasite growth Assays

Antiparasite multiplication assays were performed by co-culturing mature asexual blood stage forms of *Pf*-iRBCs with human T-2, N-C-T-2, C-T-2 or with inactivated C-T-2 used at different concentrations (0.25 to 10 μM final) for 96 h (37 °C, 5% CO_2_). Assays were done in triplicates and parasitemia was determined by microscopic examination of cells after staining blood thin smears with eosin/giemsa. Antiparasitic inhibition percentages (%) were measured as the variation in term of parasitemia between the tested condition and the control (*Pf*-iRBC). For neutralization assays, a polyclonal rabbit anti-(T-2) IgG (Thermo Scientific, ref PA5-35878) was used at a ratio of antigen:antibody of 1:10.

### Parasite multiplication inhibition tests using ADCI assays

ADCI assays were carried out by coculturing 2.10^5^ CD14-CD16 enriched human MNs with IgG (used at 2 mg/ml final concentration) from either malaria immune (PIAG a pool of Immune African Gamma Globulins) or naive (nIG) donors, to which synchronised *Pf*-iRBCs (0.5% starting parasitemia and 2.5% hematocrit) were added. Assays were performed in a 48-wells sterile culture plate for 96 h at 37 °C in a CO_2_ incubator (5% CO_2_ atmosphere). At 48 and 72 hours, 50 μl of complete medium were added to each well. After 96 hours the assay was stopped and the parasitemia determined by flow cytometry (FACSCalibur, BD Biosciences, CA).

Flow cytometry enumeration of infected erythrocytes with viable malaria parasites was performed by double staining of DNA and RNA using hydroethidine (HE) and thiazole orange (TO) (Sigma-Aldrich Corp.) as described elsewhere. The specific growth inhibitory index (SGI) was calculated according to the following formula: SGI = 100 × [1−(percent parasitemia with MN and test IgG/percent parasitemia with test IgG)/(percent parasitemia with MN and naïve IgG/percent parasitemia with naïve IgG)].

100 μl of ADCI supernatant were collected in duplicate wells after 8, 24, 48 or 96 h, and kept at −20 °C until use for T-2 ELISA measurement. As a positive control for the ELISA, the supernatant from MNs stimulated with 0.2 μg/ml of LPS (*Escherichia coli* 0127:B8, from Sigma) was used for T-2 assessment.

### Parasite and T-2 Immunolocalisation assays

After preparation of PVM-enclosed merozoite structures (merozoites “bags”) from which the egress of malaria parasites was checked every 1–2 hrs, they were incubated for 2 h with 50 μg/ml of recombinant human T-2 (Sigma). A blood thin smear was then performed, fixed and permeabilized with acetone/methanol (50/50). Rabbit anti-T-2 IgG (Santa Cruz, at 1:50 dilution) was then added, and incubated for 1 hour in a wet chamber (37 °C, 5% CO_2_). The negative control consisted of a non-relevant rabbit IgG, specific of liver stage antigen preparation, kindly provided by Dr. K. Brahimi (Institut Pasteur). In parallel, other blood thin smears were performed, with the relevant antibody controls (to check for the specificity of binding of either PIAG IgG or T-2 to merozoite structures).

For T-2 localization, the secondary antibody was a goat anti-rabbit IgG coupled with Alexa 488 (Molecular Probes) and used at 1:500 dilution. Parasite nuclei were visualized by staining with a solution of PI (Sigma) before mounting the glass slides. Images were acquired using an inverted laser-scanning confocal microscope (LSM 510, Zeiss).

### Serum creatine kinase and alanine aminotransferase measurements

Alanine Aminotransferase (ALT) activity was measured from mouse serum using ALT- activity assay kit (Sigma) following manufacturer’s instructions. The ALT activity is determined by a coupled enzyme assay, which results in a fluorogenic product (λex = 535 nm; λem = 587 nm), proportional to the pyruvate generated. ALT activity is reported as nmol/min/mL = mU/mL where one unit (U) is defined as the amount of enzyme that generates 1 μmole of pyruvate per minute at 37 °C. Creatine Kinase (CK) activity was measured from mouse serum using CK-activity assay kit (Ray Biotech) following manufacturer’s instructions. The CK activity is determined by a coupled enzyme assay, which results in a colored product with strong optical density at 450 nm. CK activity is reported as nmol/min/mL = mU/mL where one unit (U) of CK is the amount of enzyme that will generate 1.0 μmol of NADH per minute at pH 9.0 at 37 °C.

### Statistical analysis

Significant differences in mice survival were determined by generating Kaplan–Meier plots and log-rank analysis. When different populations or groups were tested, normally distributed data were analyzed by ANOVA or the Student unpaired *t* test. When data were not normally distributed, nonparametric tests (Kruskal-Wallis or Mann-Whitney tests) were used. Data were considered to be significantly different when the *P* value was <0.05.

## Additional Information

**How to cite this article:** Roussilhon, C. *et al*. The antimicrobial molecule trappin-2/elafin has anti-parasitic properties and is protective *in vivo* in a murine model of cerebral malaria. *Sci. Rep.* **7**, 42243; doi: 10.1038/srep42243 (2017).

**Publisher's note:** Springer Nature remains neutral with regard to jurisdictional claims in published maps and institutional affiliations.

## Supplementary Material

Supplementary Information

## Figures and Tables

**Table 1:** Quantification of tissue cytokine transcripts after sequential i.p Ad-vectors and *Pb*ANKA infection (D2 and D5).

Median Rq	Day 2		p values	Day 5		p values
	i.p Ad-null + *Pb-*ANKA mice (n = 6)	i.p Ad-T-2 + *Pb-*ANKA mice (n = 8)		i.p Ad-null + *Pb-*ANKA mice (n = 6)	i.p Ad-T-2 + *Pb-*ANKA mice (n = 5)	
Spleen-TNFα	0.922	*0.159*	0.0087	0.896	1.44	0.0823
Spleen-IL1β	0.755	*0.234*	0.0087	0.99	1.21	NS
Spleen-IL6	0.612	*0.042*	0.0043	1.22	1.55	NS
Spleen-IL10	1.111	0.55	NS	0.856	*0.485*	0.0173
Spleen-IFNγ	1.048	*0.136*	0.0087	1.066	0.757	NS
Spleen-MIP-1α	2.265	1.135	NS	0.95	0.883	NS
Spleen-MIP-2	1.007	0.449	NS	ND	ND	NA
Spleen-MCP1	0.596	*0.119*	0.0087	ND	ND	NA
Spleen-KC	1.061	*0.177*	0.0087	0.94	1.417	NS
Lung-TNFα	1.048	*0.173*	0.0043	0.969	**3.556**	0.0043
Lung-IL1β	0.883	1.199	NS	1.044	**2.82**	0.0043
Lung-IL6	0.659	0.556	NS	1.084	1.038	NS
Lung-IL10	1.295	**11.58**	0.0043	0.955	**1.97**	0.0303
Lung-IFNγ	0.685	0.724	NS	1.092	0.566	0.0823
Lung-MIP-1α	0.968	*0.321*	0.0173	1.143	**1.801**	0.0303
Lung-MIP-2	0.846	*0.264*	0.0519	ND	ND	NA
Lung-MCP1	0.939	*0.275*	0.0303	ND	ND	NA
Lung-KC	0,743	*0.287*	0.0087	0.941	1.361	NS
Brain-TNFα	0.515	*0.185*	0.0173	0.853	1.233	NS
Brain-IL1β	0.847	0.816	NS	1.13	1.094	NS
Brain-IL-6	0.635	0.5	NS	1.137	1.328	NS
Brain-IL10	ND	ND	NA	0.857	1.622	NS
Brain-IFNγ	0.36	*0.102*	0.0519	1.29	0.46	NS
Brain-MIP-1α	0.666	0.729	NS	0.984	1.792	NS
Brain-MIP-2	1.116	0.778	NS	0.564	3.551	NS
Brain-MCP1	0.986	1.208	NS	1.102	1.205	NS
Brain -KC	1.494	0.16	0.0823	1.312	1.407	NS
Liver-TNFα	0.742	2.858	0.0823	1.037	**9.15**	0.0079
Liver-IL1β	1.013	0.488	NS	1.074	0.32	0.0635
Liver-IL-6	0.97	0.283	NS	1.063	**62.7**	0.0043
Liver-IL10	0.996	0.926	NS	1.269	2.387	0.0952
Liver-IFNγ	0.736	1.509	NS	1.146	**7.21**	0.0079
Liver-MIP-1α	0.78	2.615	NS	1.281	2.51	NS
Liver-MIP-2	0.962	4.09	NS	0.835	6.24	NS
Liver-MCP1	0.839	**2.745**	0.0303	0.846	**16.954**	0.0043
Liver-KC	0.7	0.954	NS	0.876	0.888	NS

The same samples obtained for Fig. 23 analysis were assessed by qRT-PCR for cytokines mRNA expression at D2 and D5 (Table 1). Results are expressed as median Rq ( = 2^−∆∆Ct^), using HPRT as the normalizer gene and ‘Ad-null + *Pb*ANKA’ as the reference treatment.

For clarity, values *in italics* and **bold** in the ‘Ad-T2’ columns represent statistically significant (p < 0.05) Rq decreases (<1) and increases (>1), respectively; ND: non detected; NS: non significant.

**Table 2:** Quantification of tissue cytokine transcripts after sequential i.n Ad-vectors and i.p *Pb* ANKA infection (D2).

Median Rq	Day 2		p values
	**i.n Ad-null** + ***Pb−*****ANKA mice** (n = 6)	**i.n Ad-T-2** + ***Pb−*****ANKA mice** (n = 8)	
Spleen-TNFα	1.952	*0.24*	0.02
Spleen-IL1β	0.71	*0.26*	0.0023
Spleen-IL6	9.34	*0.1*	0.0196
Spleen-IL10	0.791	0.831	NS
Spleen-IFNγ	7.41	*0.285*	0.0022
Spleen-MIP-1α	4.39	*0.141*	0.0012
Spleen-MIP-2	0.527	*0.055*	0.0176
Spleen-MCP1	5.792	*0.65*	0.0012
Spleen-KC	2.099	*0.141*	0.0012
Lung-TNFα	1.6	2.225	NS
Lung-IL1β	1.475	2.3	NS
Lung-IL6	1.111	1.392	NS
Lung-IL10	5.053	**49.6**	0.0007
Lung-IFNγ	1.804	5.02	0.0806
Lung-MIP-1α	1.505	3.623	0.0806
Lung-MIP-2	1.895	*0.58*	0.0023
Lung-MCP1	4.194	**36.97**	0.0027
Lung-KC	1.642	*0.6*	0.0221
Brain-TNFα	0.092	0.138	NS
Brain-IL1β	1.592	1.605	NS
Brain-IL-6	0.12	0.184	NS
Brain-IL10	ND	ND	NS
Brain-IFNγ	0.111	0.165	NS
Brain-MIP-1α	0.178	0.207	NS
Brain-MIP-2	0.27	0.264	NS
Brain-MCP1	0.295	0.337	NS
BrainJ2-KC	0.275	0.267	NS

The same samples obtained for Fig. 4B–D analysis were assessed for cytokine mRNA levels in spleens, lungs and brains. They were measured by RT q-PCR and expressed as median Rq ( = 2^−∆∆Ct^), as explained in Table 1 legend. Codes and statistical significance are as in Table 1.
