# Supplementary material for: The antimicrobial molecule trappin-2/elafin has anti-parasitic properties and is protective in vivo in a murine model of cerebral malaria
Source: Sci Rep. 2017 Feb 9;7:42243. doi: 10.1038/srep42243 (PMC5299836; doi:10.1038/srep42243)

Supplementary Information

The antimicrobial molecule trappin-2/elafin has anti-parasitic properties and is protective *in vivo* in a murine model of cerebral malaria

Christian Roussilhon¹, Gilles Bang¹, Fabien Bastaert^{2,3,4}, Brigitte Solhonne^{2,3,4}, Ignacio Garcia-Verdugo^{2,3,4}, Roger Peronet⁵, Pierre Druilhe⁶, Anavaj Sakuntabhai¹, Salaheddine Mecheri⁵, and Jean-Michel Sallenave^{2,3,4,★}

¹Unité de génétique fonctionnelle des maladies infectieuses and CNRS Unité de recherche associée 3012 ; Paris, 75015, France; ² Unité de Défense Innée et Inflammation, Institut Pasteur, 25 rue du Dr Roux, Paris, 75015, France ; ³ INSERM U874, Institut Pasteur ; ⁴ INSERM U1152, Faculté de Médecine site Bichat, Université Paris Diderot, Université Sorbonne Paris-Cité, 16, rue Henri Huchard, Paris, 75018, France ; ⁵ Institut Pasteur, Unité de Biologie des Interactions Hôte Parasites,⁷ CNRS ERL9195 and ⁸INSERM U1201, Paris F-75015, France, ⁶TheVac4all initiative, Institut Cochin, Paris, France.

Figure 3 is a scatter plot with error bars showing T2 levels (pg/ml) in the serum of Ad-T2-infected mice at D2 and D5. The y-axis ranges from 0 to 5000 pg/ml. The x-axis shows two time points: D2 and D5. For each time point, there are two groups: Ad-null and Ad-T2. At D2, Ad-null mice have T2 levels near 0 pg/ml, while Ad-T2 mice have significantly higher levels around 3800 pg/ml. At D5, Ad-null mice have T2 levels near 0 pg/ml, while Ad-T2 mice have significantly higher levels around 3000 pg/ml. Asterisks (*) indicate statistical significance between the Ad-null and Ad-T2 groups at both time points.

Time Point	Group	T2 Level (pg/ml)
D2	Ad-null	~0
	Ad-T2	~3800
D5	Ad-null	~0
	Ad-T2	~3000

Mice serum (see Fig 1 legend) was analysed for T-2 protein expression by ELISA. Each individual symbol represents a different mouse (only 4 Ad-T-2 mice were alive at D5 in this experiment). Levels of serum T-2 present in Ad-T-2/Ad-null mice + *PbANKA*-treated mice were compared at each time point with * indicating statistical differences (Mann-Whitney test, $p < 0.05$).

Mice serum (see Fig 1 legend) was analysed for T-2 protein expression by ELISA. Each individual symbol represents a different mouse (only 4 Ad-T-2 mice were alive at D5 in this experiment). Levels of serum T-2 present in Ad-T-2/Ad-null mice + *PbANKA*-treated mice were compared at each time point with * indicating statistical differences (Mann-Whitney test, $p < 0.05$).

Fig S2

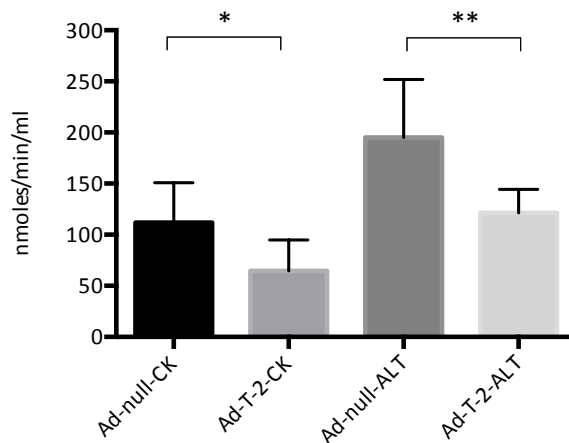

Figure S2: The level of systemic markers of cellular disturbance and liver damage (CK AND ALT) are reduced in Ad-T-2-treated mice, after *PbANKA* infection

Mice serum (see Fig 4 legend) was used to measure creatine kinase (CK) and aminotransferase (ALT) levels, two systemic markers of organ damage, using specific kits as explained in Methods. The geometric means (and 95% CI of the geometric means) of CK and ALT levels were determined, with * indicating $p=0.0313$ and ** indicating $p=0.0047$.

Fig Suppl 3 'raw data' of Fig 5

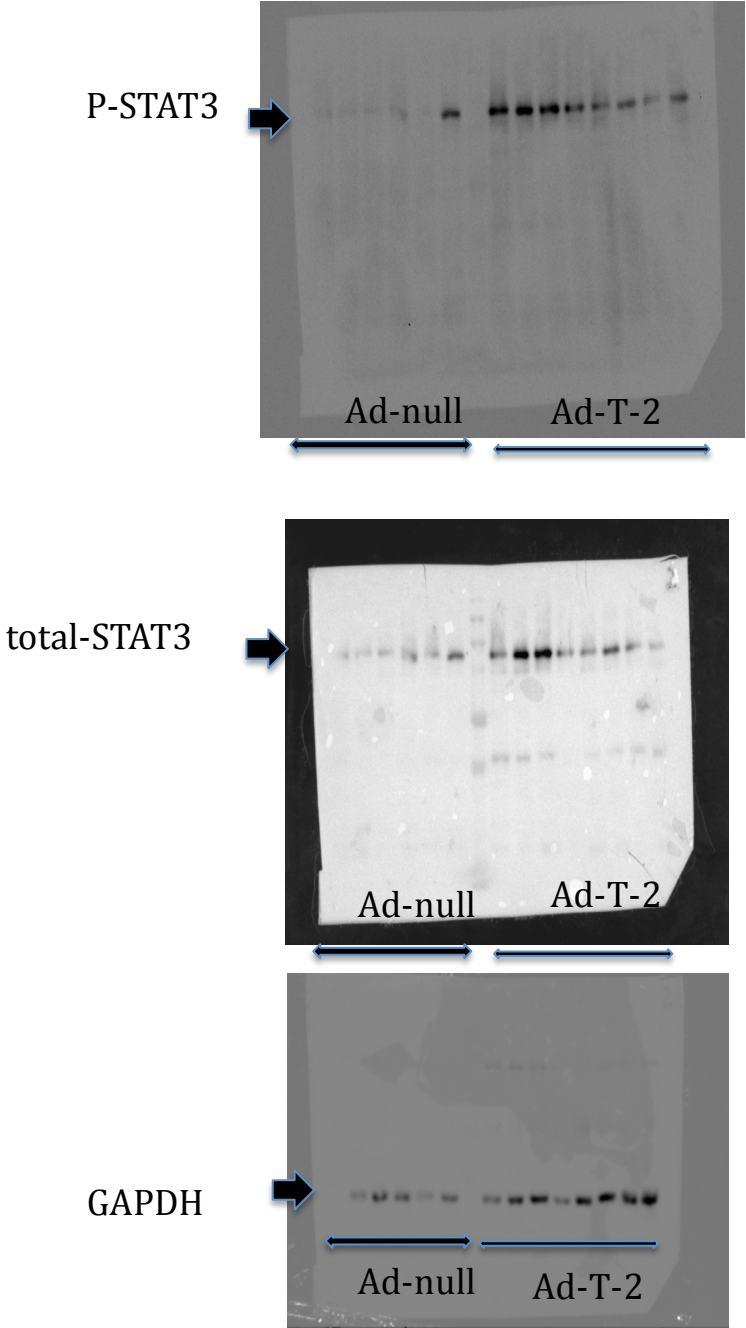

Supplement: Supplementary Information [file srep42243-s1.pdf]
